# Supplementary material for: Road side unit deployment optimization for the reliability of internet of vehicles based on information transmission model
Source: PLoS One. 2024 Dec 18;19(12):e0315716. doi: 10.1371/journal.pone.0315716 (PMC11654949; doi:10.1371/journal.pone.0315716)
Supplement: Attachment — Submitted filename: Response Letter.docx [file pone.0315716.s001.docx]

**Capital University of Economic and Business**

**Beijing, China**

**charles3000@cueb.edu.cn**

**November 2, 2024**

Dear Editors

We thank the reviewers for their generous comments on the manuscript and have edited the manuscript to address their concerns.

We add a section about Related work, focusing on the contributions of related literature and improvements of our model. Additionally, we provide more detailed descriptions of the model content and include figures of results. Furthermore, we add the description of the model's performance in different traffic scenarios.

We believe that the manuscript is now suitable for publication in PLOS One.

Dr. Guangtong Hu

Ph.D. Candidate in Capital University of Economic and Business

On behalf of all authors.

**Reviewer 1 (Ebrahim E. Elsayed)**

1. The presence of formatting issues with some equations detracts from the clarity of mathematical reasoning.

**Answer:** Thank you for your reminder. We check all the formulas and revise the format of Equations (15) and (16).

2. The description of the NNSGA-II algorithm could be enhanced by adding flowcharts or pseudocode for better understanding.

**Answer:** We add a description of the flowchart for the NNSGA-II algorithm in Figure 3.

3. A need exists for more detailed examples and visualizations to illustrate the algorithm's practical applications, with current examples lacking robustness.

**Answer:** We add visual results (Fig 5) and detailed descriptions to illustrate the practical application of the model and algorithm.

4. The model's adaptability to different road network types and limitations observed during simulations requires clarification.

**Answer:** We add the model's adaptability to different types of road networks in Section 5.2 and discuss the limitations observed during the simulation process in the Conclusion.

5. The rationale behind selecting specific parameter values for mutation and crossover probabilities needs further explanation, alongside any sensitivity analysis conducted.

**Answer:** In our study, the selection of specific mutation and crossover probability parameters is based on extensive experimental data. We conduct a wide-ranging sensitivity analysis to verify the model's sensitivity to different parameter values. Figure 9 illustrates the sensitivity analysis of mutation and crossover probabilities.

6. This paper develops a bi-objective optimization model utilizing the NNSGA-II algorithm for improved RSU deployment strategies in IoV systems. The effective comparison of the proposed model with benchmark methods highlights notable advantages in specific scenarios. The need for improvements in mathematical clarity, inclusion of visual aids, and detailed examples is crucial. Insights into parameter selection and model adaptability to various road types are encouraged. With these enhancements, the work is positioned to yield significant insights into practical applications in smart transportation systems.

**Answer:** We have addressed the reviewer's comments by improving mathematical clarity, adding visual aids and detailed examples, and providing insights into parameter selection and model adaptability to various road types. These enhancements significantly strengthen the practical applicability and robustness of our work.

7. The necessity exists for a detailed simulation comparison to elucidate the proposed model's performance. Further exploration of the model's algorithms is essential for bolstering claims of efficiency.

**Answer:** We demonstrate the performance of the proposed model in different traffic scenarios compared to other models in Section 5.2, showcase the superior performance of the proposed algorithm through comparisons in Section 5.3, and present the results of sensitivity analysis for various parameters in Section 5.4.

8. Expanding evaluation breadth to encompass various scenarios enhances robustness.

**Answer:** We compare six different traffic states, ranging from smooth to extremely congested, in Section 5.2.

9. Increasing figure size is crucial for improved interpretation of key data and trends.

**Answer:** Thank you for your reminder. We increase the size of all figures.

10. The adaptation of the model to distinct environmental scenarios warrants exploration.

**Answer:** We compare six different traffic states, ranging from smooth to extremely congested, in Section 5.2.

11. Conducting a thorough cost-benefit analysis is critical for understanding economic impacts. An additional explanations or examples of the applied NSGA-II algorithm could help non-expert readers understand its implementation and significance.

**Answer:** We add a discussion on the cost-benefit analysis and socioeconomic impacts of RSU deployment optimization in the introduction section. In Section 4, we provide additional descriptions and explanations of the NNSGA-II algorithm.

12. While numerical results are mentioned, consider providing more details about the test scenarios, conditions, and parameters used in the simulations to enhance reproducibility and understanding of the results.

**Answer:** We add details about the test scenarios, conditions, and parameters used in the simulations in Section 5.1.

**Reviewer 2 (Anonymous)**

1. Introduction should cover the background of the area, problem motivation, importance of problem, methodology used, results analysis and concluding remarks in a short note.

**Answer:** We add the results analysis and conclusions in the introduction section.

2. The authors should include a section for literature survey (Section II- Related work).

**Answer:** Thank you for your reminder. We add the Section II - Related work.

3. Authors should very clearly state how does your work advances the state of the art. At the end of Section II, the authors should give a brief summary of related work to show the difference and improvement of this work. Maybe including a table summarizing the related work could help identifying gaps that your work fills.

**Answer:** We summarize the related work and describe the improvements of our work at the end of Section 2. Additionally, we add a table to highlight the limitations of related work and our solutions.

4. I suggest to keep a table at the end of section II.

**Answer:** In Section 2, we add a table to highlight the limitations of related work and our solutions.

5. Section II may be divided into subsections like conventional methods, cooperative methods, non-cooperative methods, online/offline methods and their merits and demerits.

**Answer:** We categorize the related literature into four different types: Integer Programming, Heuristic Algorithms, Mathematical Modeling and Optimization Algorithms, Hybrid Management Strategies.

6. The simplicity of Figure 1 is noted, and it is suggested that more details be incorporated to depict the communication between CVs, and RSUs. This addition would provide greater clarity to the reader regarding the entire system model.

**Answer:** Thank you for your reminder. We re-draw Figure 1, and adding more details.

7. This submission lacks a comprehensive discussion of pertinent works, notably omitting references to key sources such as: a. "Contact duration-aware cooperative cache placement using genetic algorithm for mobile edge networks." Computer Networks 193 (2021): 108062. b. Service Caching and User Association in Cache Enabled Multi-UAV Assisted MEN for Latency-Sensitive Applications.” Computers and Electrical Engineering (Elsevier) (2024)

**Answer:** We conduct a thorough review of the literature and include citations in Section 5.
